# Social support and demoralization among patients with lung cancer in China: A moderated mediation model of self-efficacy and symptom distress

**DOI:** 10.1016/j.apjon.2025.100749

**Published:** 2025-06-30

**Authors:** Zhiheng Ping, Chunyan He, Shuhui Liu, Rong Tan, Deying Hu

**Affiliations:** a Department of Nursing, Union Hospital, Tongji Medical College, Huazhong University of Science and Technology, Wuhan, China; b School of Nursing, Tongji Medical College, Huazhong University of Science and Technology, Wuhan, China; c Cancer Center, Union Hospital, Tongji Medical College, Huazhong University of Science and Technology, Wuhan, China

**Keywords:** Demoralization, Social support, Self-efficacy, Symptom distress, Lung cancer

## Abstract

**Objective:**

To investigate whether the relationship between social support and demoralization in Chinese patients with lung cancer is mediated.

**Methods:**

Between January and June of 2024, a comprehensive cross-sectional investigation was carried out at three top-tier hospitals in Hubei Province, China. A grand total of 418 individuals diagnosed with lung cancer participated in the research by filling out confidential self-assessment questionnaires. To gauge the mediating and moderating influences, the bootstrapping technique was applied for regression analysis.

**Results:**

Among 418 patients with cancer, demoralization was negatively correlated with both social support (*ρ* ​= ​0.638, *P* ​< ​0.01) and self-efficacy (*ρ* ​= ​-0.749, *P* ​< ​0.01). Social support significantly predicted self-efficacy (B ​= ​0.334, *P* ​< ​0.01), which in turn was strongly associated with reduced demoralization (B ​= ​-1.130, *P* ​< ​0.001). Additionally, symptom distress moderated the effect of social support on self-efficacy (B ​= ​-0.003, *P* ​< ​0.05), with higher distress weakening the positive impact of social support on self-efficacy.

**Conclusions:**

Social support plays a crucial role in enhancing self-efficacy, which subsequently contributes to the reduction of demoralization. However, it is important to note that symptom distress significantly undermines these beneficial effects, leading to heightened feelings of helplessness and hopelessness.

## Introduction

Lung cancer cases continue to rise globally year after year, solidifying its status as the most prevalent and lethal form of cancer. It consistently ranks at the top for both new diagnoses and cancer-related fatalities worldwide.^1^^,^^2^ However, owing to the insidious onset of lung cancer and the absence of specific symptoms in its early stages, the majority of patients are diagnosed at an advanced stage, thereby missing the opportunity for potentially curative surgical intervention.^3^ Thus, upon receiving a diagnosis of lung cancer, patients encounter a considerable psychological burden stemming from the threat to their lives. They may experience a spectrum of emotions, including depression, sadness, helplessness, frustration, and even a profound loss of will and hope for survival. This emotional state was termed “demoralization” by American psychiatrist Frank in 1996.^4^

Demoralization is a common psychological issue among cancer patients,^5^ characterized by a mental state in which individuals struggle to cope with chronic stress. This condition primarily manifests as feelings of helplessness, hopelessness, and a profound sense of meaninglessness.^6^^,^^7^ Numerous studies have demonstrated that demoralization can lead to several negative consequences for patients,^8^ including a deterioration in quality of life,^9^^,^^10^ overall well-being, and an increase in suicidal ideation.^11^ A systematic review has demonstrated that patients with lung cancer exhibit a significantly higher prevalence of demoralization syndrome, ranging from 33% to 87.5%,^12^ compared to other cancer populations, where the prevalence is between 24% and 36%.^13^ In recent years, the incidence has been on the rise.^14^ This condition is associated with adverse outcomes such as sleep disorders,^14^^,^^15^ depression,^16^ anxiety,^16^ existential distress,^17^^,^^18^ and death anxiety,^12^ and it shows a strong correlation with suicidal ideation,^13^^,^^14^ affecting up to 71.4% of patients,^19^ while the prevalence of depression is around 50%.^20^ Therefore, addressing demoralization in patients with lung cancer is of urgent importance.

Symptom distress denotes the physical and emotional discomfort patients experience due to illness-related or treatment-induced symptoms.^21^ Currently, the primary treatment methods for patients with lung cancer include a combination of surgery, chemotherapy, radiation therapy, biological therapy, and additional therapeutic approaches.^22^ During the treatment process, patients may encounter a range of symptoms. The most prevalent among these are physical manifestations, including fatigue, pain, and hair loss^23^ and as well as characteristic symptoms such as dyspnea.^24^ These symptoms are often interrelated and can interact with one another, potentially exacerbating certain manifestations and resulting in considerable physical and psychological distress for patients.^18^^,^^25^ Mounting data links ongoing symptom distress to a notably elevated demoralization risk in cancer patients.^26^^,^^27^ And symptom distress may dynamically modulate the role of protective resources.^28^

Social support encompasses the tangible and psychological aid individuals obtain from family, peers, or community amidst stress situations.^29^ It serves as a vital resource for physical recovery by providing essential information and financial aid. Evidence shows social support strongly shields against demoralization.^30^ Patients with lung cancer benefiting from increased social backing are more apt to gain knowledge about their condition and to receive support from their family, friends, and the community. This support fosters a sense of being cared for and valued, which in turn enhances their psychological recovery and reduces stress levels, thereby mitigating feelings of demoralization.^31^^,^^32^ To truly foster healthy emotional expression, we need a village—think supportive families, understanding workplaces, and a society that gets it. Drawing from Supportive Care theory,^33^^,^^34^ it's clear that when we're talking about cancer patients, their needs go way beyond just the physical. They're looking for information, emotional support, social connection, and someone to help them navigate the psychological minefield. It underscores the significance of delivering continuity of care that is customized to meet patients' specific needs. Social support, serving as an external lifeline, can strengthen the emotional resilience of cancer patients, acting as a protective barrier against feelings of demoralization.^35^ Its mechanisms are in accordance with the fundamental principles of supportive care theory.

Self-efficacy is defined as an individual's confidence in their capacity to effectively manage the health challenges and treatment requirements associated with cancer.^36^ Cancer survivors must continuously engage in self-management of their cancer-related challenges and treatment side effects to maintain or restore their overall health and well-being.^37^^,^^38^ Research has demonstrated that, similar to social support, self-efficacy is regarded as a crucial protective factor against demoralization.^27^ For individuals to express their emotions in a healthy way, they need strong psychological backing from their loved ones, professional circles, and the broader community. According to social cognitive theory,^39^ cancer patients require comprehensive assistance that addresses not just their physical symptoms but also their need for accurate information, emotional comfort, meaningful social connections, and mental well-being. Studies have demonstrated that social support improves self-efficacy, thereby promoting health behaviors.^40^ Patients with high self-efficacy are able to regulate anxiety and depressive emotions through positive self-talk (e.g., “I can find ways to relieve the pain”), preventing these emotions from escalating into demoralization.

The conservation of resources theory^28^ posits that people manage stress by actively securing and safeguarding key assets—such as social connections and personal confidence—since the depletion or potential loss of these resources often lies at the heart of emotional strain. Essentially, well-being hinges on preserving what matters most, while instability in these areas fuels psychological turmoil. Social support (emotional, informational, and instrumental support) is a primary resource that patients obtain from their external environment to help them cope with the stress of illness. Additionally, social support can be transformed into secondary resources (self-efficacy) through means such as role modeling and verbal persuasion. However, patients experiencing severe symptom distress, due to limitations in physiological function, such as dyspnea and pain or treatment side effects, such as fatigue, face continuous resource depletion. This may diminish the efficiency of social support in transforming into self-efficacy, leading to a vicious cycle of resource loss, decreased self-efficacy, and exacerbation of demoralization.

Therefore, tackling demoralization by tapping into social support as an outside boost and self-efficacy as an inner strength is really something we need to get on top of. However, research tends to look at social support and self-efficacy as separate entities impacting demoralization, missing the bigger picture of how they might work together in a kind of chain reaction. Research indicates that strong social support networks can help mitigate feelings of hopelessness in patients by fostering a sense of empowerment—particularly by boosting their self-assurance in handling their condition. However, the effectiveness of this psychological shift largely depends on how advanced the illness has become. Based on the conservation of resources theory, the purpose of this study is to explore the relationship between social support and demoralization in patients with lung cancer, the mediating role of self-efficacy and symptom distress. This approach establishes a foundation for personalized mental health support, aligning the intensity of interventions with the degree of an individual's emotional distress. The objective is to address demoralization at its inception and prevent it from escalating into more severe consequences, such as suicide.

In the context of lung cancer, various psychological and social factors may interact and influence patients' mental health outcomes. To establish a foundation for our hypotheses, we conducted preliminary analyses to examine the relationships between key variables in this study. The analysis indicated that there were significant correlations among social support, self-efficacy, symptom distress, and demoralization, which guided the formulation of our hypotheses.

**Fig. 1:**
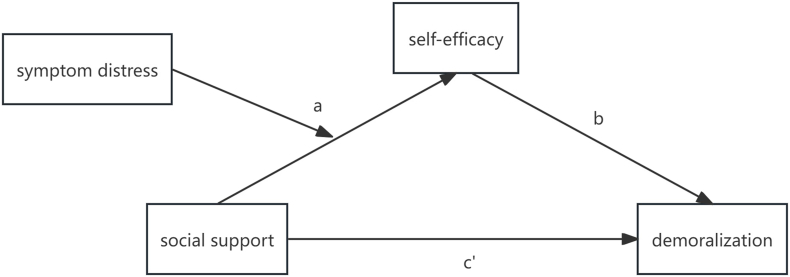
The hypothesized moderated mediation model.

Based on the Conservation of Resources theory and previous research, the following hypotheses are postulated (Fig. 1).

H1 Social support is negatively correlated with demoralization in patients with lung cancer.

H2 Self-efficacy mediates the relationship between social support and demoralization.

H3 Symptom distress moderates the relationship between social support and self-efficacy.

## Methods

### Participants and recruitments

Patients diagnosed with lung cancer and seeking treatment at three reputable 3A hospitals in Wuhan, China, from January to June of 2024, took part in our research. The study was conducted using a convenience sampling method, with individuals meeting these requirements being included: (1) a definitive lung cancer diagnosis through histopathological testing; (2) aged 18 or above; (3) in possession of sound cognitive ability, allowing for coherent communication and comprehension; (4) provide well-informed consent and opt in of their own volition. The exclusion criteria included: (1) unawareness of condition; (2) incapacity to engage from critical sickness; (3) past or lineage of acute psychological disorders.

Before initiating the inquiry, we thoroughly articulated the objectives and methodology of the study to the department leaders. Upon receiving their approval, we meticulously evaluated potential candidates for interviews, adhering to stringent eligibility and exclusion criteria. Ethical approval for this study was obtained from the Ethics Committee of Huazhong University of Science and Technology, Tongji Medical College (Approval No. 2024-S073). All participants were fully informed about the research goals and methods, as well as their rights—such as the confidentiality of their information and the option to withdraw from the study at any stage. Written informed consent was obtained from all participants prior to their involvement. After obtaining their informed consent, we distributed the surveys.

Throughout the questionnaire process, researchers were present to address any questions participants might have had in real-time. Once completed forms were collected, they underwent a comprehensive secondary review. We excluded any responses that were incomplete or exhibited uniform patterns across all options, including overly similar answers—thereby ensuring that our data was of high quality, accurate, and complete.

### Sample size

The research sample was determined through a priori power analysis conducted with G∗Power, adhering to a multivariate linear regression model aimed at identifying key and moderating factors. The parameters were set as follows: statistical Power (Power ​= ​0.80), significance level (*α* ​= ​0.05); A moderate effect size was used for the main effect, and a conservative small effect size was used for the moderating effect (interaction term).^41^ The results showed that 85 samples were needed for the main effect model (three predictors) and 395 samples were needed for the moderating effect model (four predictors and interaction terms). In order to ensure the rigor of the moderator effect test, the final target sample size was set as 400 cases (with an additional 5 cases to account for missing data), which met the stability requirements of complex model and Bootstrap method. This study aimed for a minimum sample size of 400 participants. We received 446 completed questionnaires, though 28 contained missing or inconsistent responses. After removing these invalid submissions, we retained 418 useable surveys—achieving an impressive 93.72% valid response rate that exceeded our initial requirements.

### Instruments

Demographic characteristics: Participants completed a two-part survey designed to gather comprehensive data. The first section captured sociodemographic details, including age, gender, marital status, educational level, household income per capita, employment status, place of residence, living arrangements, and medical insurance. The second part focused on medical history, documenting cancer progression (stage and type of lung cancer), diagnosis timeline, presence of metastases, and prior treatments such as surgical interventions, chemotherapy, radiation therapy, as well as targeted and immunotherapeutic approaches.

The Chinese version of MD Anderson Symptom Inventory, MDASI-C: The severity of symptom distress was measured by the MD Anderson Symptom Inventory.^42^ MDASI-C includes 19 items split into two sections. The first measures symptom intensity within the 24-h period before evaluation. Thirteen common symptoms, including pain, fatigue, and nausea, are included in the assessment. Each item is scored on a scale of 0–10, with 0 indicating no symptoms and 10 indicating the worst severity of the symptoms assessed. The second part evaluated the impact of symptoms on patients, including general activities, mood and work. Each item was scored from 0 to 10, with 0 indicating no effect and 10 indicating complete effect. The higher the score, the more severe the symptoms and the higher the degree of symptom distress. The Cronbach's alpha coefficient for this study was 0.822.

Medical Outcomes Study Social Support Survey - Chinese (Version), MOS–SSS–C: Social support was assessed by the Chinese version of Social Support Scale.^43^ The instrument comprises 19 items organized into four dimensions: tangible support, informational and emotional support, positive social engagement, and affectionate support. Participants rated each item on a 5-point Likert scale, reflecting the frequency with which they received various forms of support, where elevated scores correspond to greater perceived support levels. While an additional metric assessed participants' network of close friends and relatives, this subjective measure was excluded from our analysis. The Cronbach's alpha coefficient for this scale in the study was 0.960.

The Regulatory Emotional Efficacy Scale, RESE: It was originally created by Caprara and colleagues in 2008,^44^ and later adapted into Chinese by Wen's research team the following year.^45^ This assessment tool measures three key aspects of emotional self-regulation: perceived self-efficacy in managing positive emotions (POS), distress (DES), and anger (ANG). Each of these dimensions contains four specific items, bringing the total to 12 questions. Responses are recorded on a standard five-point Likert scale ranging from “strongly disagree” (1) to “strongly agree” (5), with higher cumulative scores indicating greater confidence in one's ability to regulate emotions. The Cronbach's alpha coefficient for the scale utilized in this study was measured at 0.878.

The Demoralization Scale, DS: The study employed the Demoralization Scale to measure participants' levels of demoralization. Originally developed by Kissane et al.^46^ and later adapted into Chinese by Hung et al.,^47^ the scale consists of 24 items grouped into five key domains: sense of failure, existential meaninglessness, perceived helplessness, restlessness, and emotional distress. Responses were recorded on a four-point Likert scale, yielding a total possible score between 0 and 96, where higher scores indicate more severe demoralization. The Cronbach's alpha for the scale in this study stood at a robust 0.902.

### Data analysis

For data management, we used EpiData 3.1, while IBM SPSS Statistics 26.0 (IBM Corp., Armonk, NY) handled the analytical heavy lifting. We summarized participants' baseline characteristics using standard descriptive statistics. Categorical differences in demoralization levels among Chinese patients with lung cancer were evaluated through chi-square tests. Since our variables weren't normally distributed, we turned to Spearman's rank correlation to examine connections between social support, self-efficacy, symptom distress, and demoralization. Hayes' PROCESS macro^48^ is a statistical tool used to perform mediation, moderation, and moderated mediation analyses, allowing researchers to test the direct and indirect relationships between variables, as well as the influence of moderators on these relationships. Each model in Hayes' PROCESS macro is designed for different analytical purposes: Model 1 tests simple moderation, Model 4 tests simple mediation, Model 7 tests moderated mediation, and other models account for more complex interactions, such as multiple mediators or multiple moderators, depending on the research design. To unpack the underlying mechanisms, we implemented Hayes' PROCESS macro (Model 4)^48^ to test whether self-efficacy mediated the social support-demoralization link. Then, using Model 7, we checked if symptom distress played a moderating role in this mediated pathway. All effects were validated through bias-corrected bootstrapping (5000 Bootstrap, 95% CI), with significance determined when zero fell outside the confidence interval. After standardizing the variables, we conducted simple slope analyses to probe moderation effects. Throughout these analyses, we adjusted for relevant demographic covariates. A two-tailed p-value below 0.05 marked statistical significance.

## Results

### Characteristics of the participants

Participants in the study spanned ages 18–79 years, with an average age of 59.42 and a standard deviation of 8.14. The bulk of the group was male, accounting for 64.35%. A considerable chunk of the participants had completed only junior high or less, with 64.59% falling into this category, and the majority lived in cities, with 57.89% calling urban areas home. In terms of family monthly income, over a third (36.12%) noted that their family's income per person was under 3000 yuan. When it came to lung cancer diagnosis, a disturbingly high percentage—69.14%—were at stage IV. Over three-quarters (77.75%) of the patients received chemotherapy. Certain demographics exhibited higher levels of demoralization, including women, those with a junior high education or less, those with a monthly family income per capita of less than 3000 yuan, farmers, those living in rural areas, individuals on rural residents' basic medical insurance, those living alone, patients with metastasis, those at stage IV, and those who received targeted therapy. Further details can be found in Table 1.

**Table 1:** Demographic and disease-related data and demoralization (*N* ​= ​418).

Variables	*n* (%)	Demoralization	*P*-value
Sex			
Male	269 (64.35)	41.07 ​± ​15.43	< 0.001^c^
Female	149 (35.65)	49.26 ​± ​15.29	
Marital status			
Married	402 (96.17)	43.89 ​± ​15.73	0.529
Unmarried/divorced/widowed	16 (3.83)	46.44 ​± ​19.09	
Educational level			
Junior secondary and less	270 (64.59)	46.49 ​± ​16.46	< 0.001^c^
High school/junior college	107 (25.60)	41.62 ​± ​13.46	
Bachelor and more	41 (9.81)	33.66 ​± ​12.34	
Family monthly income per capita (RMB)			
< ​3000	151 (36.12)	51.35 ​± ​15.39	< 0.001^c^
3000-5000	133 (31.82)	42.04 ​± ​14.67	
> ​5000	134 (32.06)	37.63 ​± ​14.19	
Employment status			
Employed	25 (5.98)	36.80 ​± ​11.26	< 0.001^c^
Retired	148 (35.41)	39.82 ​± ​15.01	
Farming	79 (18.90)	50.51 ​± ​15.52	
Unemployed	114 (27.27)	46.71 ​± ​15.59	
Others	52 (12.44)	43.42 ​± ​16.77	
Residence			
Rural	176 (42.11)	48.88 ​± ​15.10	< 0.001^c^
Urban	242 (57.89)	40.43 ​± ​15.47	
Living alone			
Yes	13 (3.11)	54.15 ​± ​16.86	0.019^a^
No	405 (96.89)	43.66 ​± ​15.74	
Medical insurance type			
Basic medical insurance for urban employee	160 (38.28)	38.69 ​± ​14.81	< 0.001^c^
Basic medical insurance for urban residents	63 (15.07)	42.75 ​± ​15.59	
Basic medical insurance for rural residents	190 (45.45)	49.01 ​± ​15.43	
Others	5 (1.20)	38.40 ​± ​9.79	
Cancer stage			
Stage I	7 (1.67)	35.14 ​± ​17.99	< 0.001^c^
Stage II	38 (9.09)	39.05 ​± ​17.90	
Stage III	84 (20.10)	39.44 ​± ​14.59	
Stage IV	289 (69.14)	46.17 ​± ​15.46	
Lung cancer type			
Adenocarcinoma	277 (66.27)	44.36 ​± ​16.32	0.679
Squamous cell carcinoma	73 (17.46)	42.23 ​± ​14.80	
Small cell lung cancer	48 (11.48)	45.19 ​± ​14.16	
Others	20 (4.78)	42.40 ​± ​17.34	
Time since diagnosis			
< ​3 months	129 (30.86)	44.15 ​± ​15.30	0.134
3–6 months	70 (16.75)	41.47 ​± ​15.19	
6–12 months	53 (12.68)	43.72 ​± ​17.06	
1–3 years	105 (25.12)	47.01 ​± ​15.98	
> ​3 years	61 (14.59)	41.57 ​± ​16.08	
Metastasis status			
Yes	339 (81.10)	45.20 ​± ​15.70	0.001^b^
No	79 (18.90)	38.77 ​± ​15.56	
Surgery			
Yes	95 (22.73)	41.94 ​± ​16.44	0.152
No	323 (77.27)	44.59 ​± ​15.66	
Chemotherapy			
Yes	325 (77.75)	43.53 ​± ​15.81	0.273
No	93 (22.25)	45.58 ​± ​16.01	
Radiotherapy			
Yes	164 (39.23)	44.58 ​± ​16.02	0.541
No	254 (60.77)	43.61 ​± ​15.77	
Targeted therapy			
Yes	137 (32.78)	46.25 ​± ​16.20	0.042^a^
No	281 (67.22)	42.89 ​± ​15.59	
Immunotherapy			
Yes	122 (29.19)	42.54 ​± ​16.12	0.231
No	296 (70.81)	44.58 ​± ​15.73	

M, mean; SD, standard deviation. The dependent variable in this study is the demoralization score, which is a continuous variable. For binary categorical independent variables, the independent samples *t* test is used to compute the *P*-value (e.g., gender, marital status). In cases where the independent variable is multi-categorical, One-Way ANOVA is applied to calculate the *P*-value (e.g., educational level).

a *P* ​< ​0.05.

b *P* ​< ​0.01.

c *P* ​< ​0.001.

### Correlation analysis

The results of the correlation analysis for the four key variables are displayed in Table 2. A strong inverse relationship emerged between demoralization and both social support (*ρ* ​= ​-0.638, *P* ​< ​0.01) and self-efficacy (*ρ* ​= ​-0.749, *P* ​< ​0.01). On the flip side, social support and self-efficacy were positively linked (*ρ* ​= ​0.612, *P* ​< ​0.01). Symptom distress showed modest negative associations with social support and self-efficacy (both ρ ​= ​-0.327, *P* ​< ​0.01), while maintaining a positive connection with demoralization (*ρ* ​= ​0.431, *P* ​< ​0.01). These patterns not only support our first hypothesis but also indicate that these variables warrant deeper exploration through moderated mediation analysis.

**Table 2:** Correlation analysis among variables in lung cancer patients.

Variables	Mean ​± ​SD	Social support	Self-efficacy	Symptom distress	Demoralization
Social support	65.62 ​± ​13.79	1			
Self-efficacy	37.08 ​± ​7.71	0.612^a^	1		
Symptom distress	33.57 ​± ​19.21	−0.327^a^	−0.327^a^	1	
Demoralization	43.99 ​± ​15.86	−0.638^a^	−0.749^a^	0.431^a^	1

SD, standard deviation.

a *P* ​< ​0.01.

### Testing for moderated mediation effect

In all effect analyses, gender, education level, monthly household income, occupation, residence status, living alone, medical insurance payment method, cancer stage, metastasis status, and targeted therapy were controlled as covariates. To kick things off, we used PROCESS macro Model 4 to dig into whether self-efficacy was a go-between in the link between social support and feeling down. After keeping all the other factors in check, the data (Table 3, Table 4) clearly shows that social support does, in fact, have a significant knock-on effect on demoralization through self-efficacy. Social support demonstrated a significant association with demoralization (B ​= ​-0.665; *t* ​= ​-14.133; *P* ​< ​0.001). The significant negative relationship between social support and demoralization persisted even when accounting for self-efficacy in the analysis (B ​= ​-0.288, *t* ​= ​-6.269, p ​< ​0.001). Notably, higher levels of social support strongly predicted increased self-efficacy (B ​= ​0.334, *t* ​= ​13.597, *P* ​< ​0.001), which in turn showed a robust negative association with demoralization (B ​= ​-1.130, *t* ​= ​-14.692, *P* ​< ​0.001). Examining the 95% confidence intervals revealed that neither the direct effect of social support [−0.378, −0.198] nor its indirect effect through self-efficacy [−0.457, −0.307] included zero (Table 4), indicating both pathways are statistically meaningful. These results demonstrate that social support influences demoralization both directly and indirectly by bolstering self-efficacy. The breakdown of effects shows the direct pathway explains 43.31% of the total impact, while the mediated pathway accounts for 56.69%, culminating in a substantial overall effect (total effect: B ​= ​0.665). Consequently, these findings provide clear support for Hypothesis 2.

**Table 3:** The mediating effect of self-efficacy on demoralization.

Variables	B	SE	*t*	*P*	LLCI	ULCI	*R*^2^
Outcome variable: Self-efficacy							0.623
Social support	0.334	0.025	13.597	< 0.001^b^	0.286	0.382	
Gender	−1.148	0.680	−1.690	0.092	−2.484	0.188	
Educational level	−0.845	0.546	−1.547	0.123	−1.918	0.229	
Family monthly income per capita (RMB)	0.793	0.513	1.545	0.123	−0.216	1.802	
Employment status	−0.255	0.320	−0.798	0.425	−0.884	0.374	
Residence	0.481	1.063	0.453	0.651	−1.609	2.571	
Living alone	0.027	1.795	0.015	0.988	−3.502	3.555	
Medical insurance type	0.385	0.647	0.596	0.552	−0.886	1.656	
Cancer stage	−0.341	0.700	−0.487	0.627	−1.718	1.036	
Metastasis status	−0.095	1.278	−0.075	0.941	−2.607	2.417	
Targeted therapy	−0.908	0.705	−1.289	0.198	−2.294	0.477	
Outcome variable: demoralization							0.808
Social support	−0.288	0.046	−6.269	< 0.001^b^	−0.378	−0.198	
Self-efficacy	−1.130	0.077	−14.692	< 0.001^b^	−1.281	−0.979	
Gender	4.262	1.057	4.033	< 0.001^b^	2.185	6.340	
Educational level	0.140	0.849	0.165	0.869	−1.528	1.808	
Family monthly income per capita (RMB)	−1.765	0.798	−2.213	0.028^a^	−3.333	−0.197	
Employment status	0.175	0.496	0.353	0.724	−0.780	1.150	
Residence	0.392	1.648	0.238	0.812	−2.848	3.632	
Living alone	2.326	2.781	0.836	0.404	−3.142	7.793	
Medical insurance type	0.213	1.002	0.213	0.832	−1.757	2.184	
Cancer stage	2.385	1.086	2.197	0.029^a^	0.251	4.519	
Metastasis status	1.199	1.980	0.606	0.545	−2.693	5.092	
Targeted therapy	−0.811	1.094	−0.741	0.459	−2.962	1.341	

B, unstandardized coefficients; SE, standard error; LLCI, lower limit of the 95% confidence interval; ULCI, upper limit of the 95% confidence interval.

a *P* ​< ​0.05.

b *P* ​< ​0.001.

**Table 4:** Total effect, direct effect and mediating effect.

Social support→Demoralization					
	Effect	SE	LLCI	ULCI	Proportion
Total effect	−0.665	0.047	−0.758	−0.573	
Direct effect	−0.288	0.046	−0.378	−0.198	43.31%
Indirect effect	−0.377	0.039	−0.457	−0.307	56.69%

SE, standard error; LLCI, lower limit of the 95% confidence interval; ULCI, upper limit of the 95% confidence interval.

**Fig. 2:**
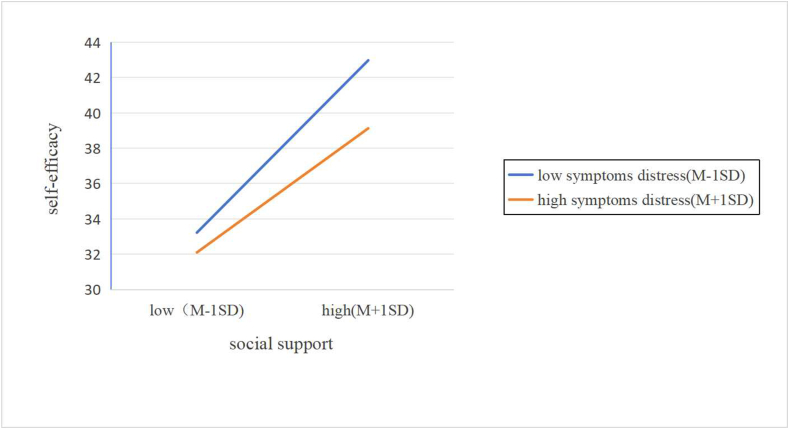
Simple slope analysis. M, mean; SD, standard deviation.

**Fig. 3:**
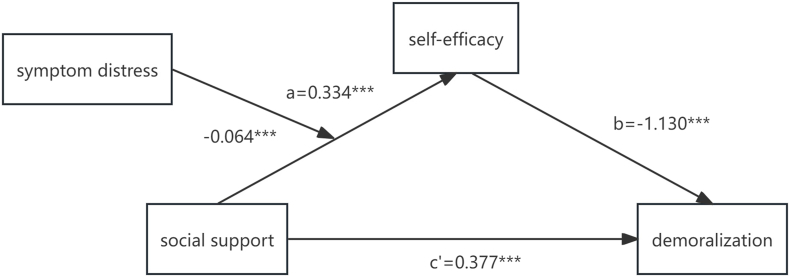
The final moderated mediation model. ∗∗∗*P* ​< ​0.001.

Secondly, Model 7 of the PROCESS macro, representing the first half of the moderation model and aligning with the hypothesized framework of this study, was employed to investigate the moderated mediation model while controlling for covariates. The results (Table 5) indicated that upon incorporating symptom distress into the model, the interaction term (social support X symptom distress) exhibited a significant moderating effect on self-efficacy (B ​= ​-0.003, *t* ​= ​-2.1978, *P* ​< ​0.05). This finding suggests that symptom distress serves as a moderator in the relationship between social support and self-efficacy. Fig. 2 illustrates the results of a follow-up simple slope analysis, which reveals an inverse relationship between symptom distress and the impact of social support on self-efficacy. Specifically, among patients with lung cancer with more severe symptoms, the role of social support in shaping self-efficacy becomes noticeably weaker than in those with milder symptoms. Essentially, the data suggests that rising levels of symptom distress gradually undermine social support's ability to predict self-efficacy. Furthermore, Table 6 showcases the subtler impacts of symptom distress across different intensity levels (M-1SD, M, M+1SD). The study reveals a notable link between symptom distress and its indirect influence on patients with lung cancer, with those experiencing less distress showing a significant effect (Table 6; effect size ​= ​0.354, 95% CI: 0.286–0.422; effect size ​= ​0.304, 95% CI: 0.254–0.355). However, for patients with greater symptom distress, this indirect effect wasn't statistically significant (Table 6; effect size ​= ​0.255, 95% CI: 0.189–0.321). This suggests that as symptom distress escalates, the likelihood of social support predicting suicidal thoughts via self-efficacy diminishes. Consequently, hypothesis 3 holds true. The complete model is depicted in Fig. 3.

**Table 5:** The moderated mediation model with self-efficacy as a mediator and symptom distress as a moderator.

Variables	B	SE	*t*	*P*	LLCI	ULCI	*R*^2^
Outcome variable: Self-efficacy							0.644
Social support	0.304	0.026	11.886	< 0.001^b^	0.254	0.355	
Symptom distress	−0.065	0.017	−3.834	< 0.001^b^	−0.098	−0.032	
Gender	−0.798	0.671	−1.189	0.235	−2.118	0.527	
Educational level	−0.743	0.536	−1.386	0.166	−1.797	0.311	
Family monthly income per capita (RMB)	0.884	0.506	1.748	0.081	−0.110	1.878	
Employment status	−0.331	0.314	−1.055	0.292	−0.949	0.286	
Residence	0.483	1.043	0.463	0.644	−1.567	2.533	
Living alone	−0.015	1.761	−0.009	0.993	−3.477	3.447	
Medical insurance type	0.345	0.635	0.543	0.588	−0.904	1.594	
Cancer stage	0.297	0.703	0.423	0.673	−1.085	1.680	
Metastasis status	0.729	1.268	0.575	0.566	−1.764	3.221	
Targeted therapy	−0.675	0.701	−0.963	0.336	−2.053	0.703	
Outcome variable: demoralization							0.808
Social support	−0.288	0.046	−6.269	< 0.001^b^	−0.378	−0.198	
Self-efficacy	−1.130	0.077	−14.692	< 0.001^b^	−1.281	−0.979	
Gender	4.262	1.057	4.033	< 0.001^b^	2.185	6.340	
Educational level	0.140	0.849	0.165	0.869	−1.528	1.808	
Family monthly income per capita (RMB)	−1.765	0.798	−2.213	0.028^a^	−3.333	−0.197	
Employment status	0.175	0.496	0.353	0.724	−0.780	1.150	
Residence	0.392	1.648	0.238	0.812	−2.848	3.632	
Living alone	2.326	2.781	0.836	0.404	−3.142	7.793	
Medical insurance type	0.213	1.002	0.213	0.832	−1.757	2.184	
Cancer stage	2.385	1.086	2.197	0.029^a^	0.251	4.519	
Metastasis status	1.199	1.980	0.606	0.545	−2.693	5.092	
Targeted therapy	−0.811	1.094	−0.741	0.459	−2.962	1.341	

B, unstandardized coefficients; SE, standard error; LLCI, lower limit of the 95% confidence interval; ULCI, upper limit of the 95% confidence interval.

a *P* ​< ​0.05.

b *P* ​< ​0.001.

**Table 6:** Conditional indirect effects of social support on self-efficacy at values of symptom distress.

Symptoms distress	Effect	SE	LLCI	ULCI
−19.210	0.354	0.035	0.286	0.422
0.000	0.304	0.026	0.254	0.355
19.210	0.255	0.034	0.189	0.321

SE, standard error; LLCI, lower limit of the 95% confidence interval; ULCI, upper limit of the 95% confidence interval.

## Discussion

This research investigates the role of social support in alleviating feelings of demoralization among Chinese patients with lung cancer, while also examining the mediating effects of self-efficacy and symptom distress. By constructing a comprehensive mediation model, this study not only elucidates the psychological mechanisms at play but also provides evidence-based strategies aimed at enhancing emotional resilience within this vulnerable population. The findings highlight significant practical implications for improving mental health outcomes through targeted psychosocial interventions.

### Social support and demoralization

The findings of this study underscore the significant role of social support in mitigating demoralization among Chinese patients with lung cancer. Our results are consistent with previous research, which highlights a robust relationship between higher levels of social support and increased resilience.^27^^,^^49^ When patients receive comprehensive social support—whether emotional, material, or informational—they are more likely to feel respected, understood, and valued, which fosters a proactive coping strategy and a sense of empowerment. This support system not only helps buffer the negative psychological impacts of the illness but also bolsters patients' ability to confront demoralization throughout their treatment.

Research indicates that insufficient social backing may amplify feelings of solitude, thereby heightening psychological discomfort and hindering adherence to therapy. As Hyland and colleagues point out,^50^ when patients lack proper emotional backing, practical resources, and clear guidance, it can severely undermine their feeling of connection. This deficit often erodes their drive and ability to stick with treatment regimens. In addition, persistent psychosocial stress can disrupt autonomic nervous system functions, impair endocrine and immune responses, and reduce the effectiveness of cancer treatments.^51^ Social support, therefore, acts as a crucial protective resource, buffering external stressors and mitigating their negative effects on both physical and mental health. During chemotherapy, support from family, peers, and health care providers has been identified as a significant source of comfort, helping patients navigate the challenges posed by their condition. Our study aligns with previous research,^52^ suggesting that a strong social support network not only reduces the levels of demoralization but also fosters better psychological adjustment and health outcomes during treatment.

This highlights the necessity for health care professionals to actively collaborate with families, social organizations, and community services to establish a robust, multi-layered support system. Strengthening such networks can enhance patients' psychological resilience, improve their quality of life, and support their overall well-being during the treatment process.

### The mediating effect of self-efficacy

Our findings indicate that social support negatively affects demoralization in patients with lung cancer through the mediating role of self-efficacy. This aligns with Bandura's Social Cognitive Theory and further validates the mechanism through which social support indirectly influences demoralization by enhancing self-efficacy.^39^ Self-efficacy is particularly important in the context of lung cancer treatment, as it determines how patients cope with the physical and psychological challenges they encounter during treatment.^27^ In this context, higher levels of social support can enhance patients' self-efficacy, which, in turn, reduces the occurrence of demoralization. When patients receive emotional support, material assistance, and informational support from family, friends, and health care teams, their self-efficacy is boosted, making them feel more capable of coping with treatment challenges. As a result, they experience reduced feelings of helplessness and hopelessness, effectively lowering the level of demoralization. Drawing from Conservation of Resources Theory, external social support mitigates stress/anxiety and bolsters health management self-efficacy. This not only helps patients maintain a positive outlook throughout long-term treatment but also increases the likelihood of treatment adherence, thereby reducing the development of demoralization. Patients with lung cancer who possess strong self-efficacy tend to approach their treatment with greater confidence and a more positive outlook. This mindset helps them navigate the difficulties of their medical journey with resilience, resulting in a diminished sense of overwhelm.^53^ With reduced stress levels, they're better positioned to embrace positive lifestyle adjustments—whether it's prioritizing mental well-being, seeking emotional support, or making healthier choices—all of which contribute to maintaining their morale and preventing discouragement. Additionally, patients with high self-efficacy typically demonstrate strong self-management skills, which enable them to better control the treatment process, improve treatment adherence, and enhance treatment outcomes. These factors collectively contribute to a reduction in demoralization levels, providing a solid psychological foundation for maintaining long-term treatment adherence and recovery.

Future studies might delve into the potential of boosting self-efficacy to alleviate demoralization among patients with lung cancer. For example, interventions such as cognitive behavioral therapy and emotion regulation training could be examined to help patients increase their self-efficacy, thereby improving treatment adherence and strengthening their resolve and resilience when facing challenges during treatment.

### The moderating effect of symptom distress

Our findings confirm a clear inverse relationship between symptom distress and self-efficacy, aligning with prior research,^54^ and validate Hypothesis 3. This investigation reveals that inflammatory markers like TNF-α and IL-6—triggered by chemotherapy—as well as hormonal fluctuations intensify psychological distress, including anxiety, depressive symptoms, and social isolation. These physiological responses ultimately undermine patients' ability to benefit from external support systems.^55^ Moreover, a direct link exists between symptom severity and feeling demoralized, aligning with findings from past research.^16^ When patients experience pain and discomfort, they often resort to negative coping mechanisms, which in turn diminishes their hope. When patients face more severe symptom distress, more attention is paid to physical discomfort. At this time, even abundant social support may have a limited effect on enhancing their self-efficacy, which in turn can affect their level of demoralization. According to the Conservation of Resources Theory, when individuals face significant symptom distress, their psychological and physiological resources are heavily depleted, reducing their ability to cope with challenges.^56^ In this scenario, despite the provision of social support, patients may still experience demoralization, particularly during the high-pressure periods of treatment. Therefore, relying solely on social support may not be sufficient to alleviate demoralization in patients with severe symptom distress. Future research could explore how to implement tiered psychological interventions based on the severity of symptom distress. For patients with mild symptom distress, focusing on enhancing social support may be an effective strategy to mitigate demoralization. However, for patients with more severe symptom distress, merely increasing social support may not be as effective. In these cases, enhancing patients' self-efficacy may be necessary to reduce the level of demoralization. These findings provide valuable guidance for future intervention strategies, particularly when dealing with patients who experience significant symptom distress, where more comprehensive support measures are needed.

### Limitations and future directions

This study certainly has several limitations that require attention. Firstly, the cross-sectional design, while suggesting potential connections, does not adequately establish causal relationships among these four factors. To thoroughly investigate these possible causal links, longitudinal studies would be more appropriate. Additionally, we employed a convenience sample, which implies that our findings may not accurately represent the broader population. Future research utilizing random sampling could provide a more representative overview. Moreover, variables such as anxiety and depression, coping mechanisms related to illness, and even positive psychological states may play subtle yet significant roles in this model. We did not incorporate these factors in our current analysis; however, it would be beneficial for future studies to include them in their investigations.

### Implications for nursing practice and research

This research provides significant insights that may influence the approach mental health professionals take toward addressing demoralization in individuals facing lung cancer. Importantly, the findings underscore the vital role of both emotional support networks and personal resilience in empowering patients to effectively manage their illness and adhere to therapeutic regimens. These revelations pave the way for the development of more targeted psychological support strategies. Throughout the course of lung cancer treatment, patients often face immense physical, emotional, and psychological stress. Social support not only provides emotional comfort but also helps patients better manage the challenges of treatment by enhancing their self-efficacy, thereby reducing the occurrence of demoralization. This finding provides valuable insights for health care providers and mental health professionals, highlighting the importance of focusing on patients' social support networks and actively promoting the enhancement of self-efficacy during treatment.

In addition, symptom distress is a crucial factor influencing demoralization in patients with lung cancer. Research shows that symptom distress during the treatment process can exacerbate feelings of helplessness and hopelessness. Therefore, future interventions should not only focus on providing social support but also incorporate strategies to manage the patient's symptoms, helping to alleviate the negative impacts of treatment. By reducing symptom distress and enhancing patients' self-efficacy, it is possible to effectively lower the incidence of demoralization, improve treatment adherence, and promote overall physical and mental well-being.

## Conclusions

This study highlights the complex interplay between social support, self-efficacy, symptom distress, and demoralization in patients with lung cancer. The findings suggest that social support plays a crucial role in enhancing self-efficacy, which in turn helps reduce the occurrence of demoralization. However, symptom distress emerges as a significant factor that can hinder the positive effects of social support and self-efficacy, exacerbating feelings of helplessness and hopelessness. These results underscore the importance of comprehensive interventions that not only enhance social support and self-efficacy but also address symptom distress. By managing symptom distress and fostering self-efficacy, health care providers can help minimize demoralization, improve treatment adherence, and support the overall well-being of patients with lung cancer.

## CRediT authorship contribution statement

**Zhiheng Ping:** Conceptualization, Methodology, Writing – Original Draft, Writing – Review & Editing. **Chunyan He:** Writing – review & editing, Data curation, Formal analysis. **Shuhui Liu:** Investigation, Software, Validation. **Rong Tan:** Resources, Supervision, Funding acquisition. **Deying Hu:** Project administration, Writing – review & editing, Supervision. All authors have read and approved the final manuscript.

## Ethics statement

This research was conducted in accordance with the guidelines set forth by the Declaration of Helsinki and received approval from the Ethics Committee of Huazhong University of Science and Technology, Tongji Medical College (Approval No. 2024-S073). All participants provided written informed consent.

## Data availability

The data supporting the findings of this study are available from the corresponding author, DH, upon reasonable request.

## Declaration of generative AI and AI-assisted technologies in the writing process

No artificial intelligence tools or services were utilized in the preparation of this work.

## Funding

This study was funded by the Natural Science Foundation of Hubei Province (Grant No. 2025AFB584). The funders played no role in the design of the study, nor in the decision-making process regarding data collection, analysis, interpretation, report writing, or submission of the article for publication.

## Declaration of competing interest

The authors affirm that there are no conflicts of interest to disclose.
